# A shift from travel-associated cases to autochthonous transmission with Berlin as epicentre of the monkeypox outbreak in Germany, May to June 2022

**DOI:** 10.2807/1560-7917.ES.2022.27.27.2200499

**Published:** 2022-07-07

**Authors:** Regina Selb, Dirk Werber, Gerhard Falkenhorst, Gyde Steffen, Raskit Lachmann, Claudia Ruscher, Sarah McFarland, Alexander Bartel, Lukas Hemmers, Uwe Koppe, Klaus Stark, Viviane Bremer, Klaus Jansen, on behalf of the Berlin MPX study group

**Affiliations:** 1 Unit 'HIV/AIDS, STI and Blood-borne Infections', Department of Infectious Disease Epidemiology, Robert Koch Institute, Berlin, Germany; 2 State Office for Health and Social Affairs (SOHSA), Unit for Surveillance and Epidemiology of Infectious Diseases, Berlin, Germany; 3 Unit 'Gastrointestinal Infections, Zoonoses and Tropical Infections', Department of Infectious Disease Epidemiology, Robert Koch Institute, Berlin, Germany; 4 Postgraduate Training in Applied Epidemiology (PAE) Unit 'Infectious Disease Epidemiology, Crisis Management, Outbreak Investigations and Training Programmes, Focal Point for the Public Health Service', Department of Infectious Disease Epidemiology, Robert Koch Institute, Berlin, Germany; 5 European Programme for Intervention Epidemiology Training (EPIET), European Centre for Disease Prevention and Control (ECDC), Stockholm, Sweden; 6 Members of the Berlin MPX study group are listed under Acknowledgements

**Keywords:** monkeypox, MPX, outbreak, MSM, men who have sex with men

## Abstract

By 22 June 2022, 521 cases of monkeypox were notified in Germany. The median age was 38 years (IQR: 32–44); all cases were men. In Berlin, where 69% of all cases occurred, almost all were men who have sex with men. Monkeypox virus likely circulated unrecognised in Berlin before early May. Since mid-May, we observed a shift from travel-associated infections to mainly autochthonous transmission that predominantly took place in Berlin, often in association with visits to clubs and parties.

Since May 2022, an international monkeypox (MPX) outbreak is ongoing, involving several countries in Europe and beyond [1]. By 22 June 2022, Germany has become one of the most affected countries worldwide, with the second highest overall number of MPX cases in Europe. In this report, we describe the epidemiology of MPX in Germany and the shift from travel-associated to autochthonous transmission in Berlin, the epicentre of the outbreak in Germany.

## Epidemiological situation in Germany

By 22 June 2022, a total of 521 laboratory-confirmed cases of MPX were reported to local public health departments (LPHA) and electronically submitted via state health departments to the Robert Koch Institute (RKI) according to the German case definition (Table 1) [2]. The first case was notified on 20 May 2022. All cases were men between 20 and 67 years of age, the median age was 38 (interquartile range (IQR): 32–44). Hospitalisation status was reported for 455 cases of which 38 cases (8%) were admitted to hospital. All 349 cases with available information on the likely mode of transmission reported sexual or other intimate contact with other men. Cases were notified by 14 of the 16 German federal states with the highest incidence in Berlin (Table 1). Cases were concentrated in urban areas and besides Berlin, notifying 69% of cases, the cities of Cologne, Hamburg, Munich, Dusseldorf and Stuttgart notified further 18% of cases (n = 91).

**Table 1:** Number, percentage and incidence of notified MPX cases by federal state and in Germany overall, 20 May─22 June 2022 (n = 521)

Federal state	Cases		Incidence (cases per 100,000 inhabitants)
	N	%	
Berlin	358	69	9.77
Hamburg	23	4	1.24
Brandenburg	9	2	0.36
North-Rhine Westphalia	59	11	0.33
Hesse	16	3	0.25
Bavaria	27	5	0.21
Saarland	2	0.4	0.20
Saxony-Anhalt	3	0.6	0.14
Baden-Wuerttemberg	13	3	0.12
Lower Saxony	5	1	0.06
Saxony	2	0.4	0.05
Rhineland-Palatinate	2	0.4	0.05
Thuringia	1	0.2	0.05
Schleswig-Holstein	1	0.2	0.03
**Germany**	**521**	**100**	**0.63**

### Symptom onset dates and probable places of infection

**Figure 1:**
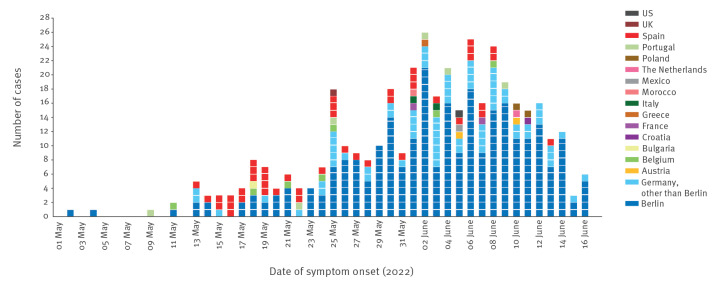
Laboratory-confirmed monkeypox cases by date of symptom onset and probable place of infection, Germany, 20 May─22 June 2022 (n = 408)

UK: United Kingdom; US: United States.

Staff of the local public health authorities (LPHA) in Germany routinely interviewed all MPX cases regarding symptom onset and probable places of infection (PPOI). Date of symptom onset and PPOI was available for 408 cases (Figure 1). Between 2 May and 22 May 2022, half of the notified cases (26/52) reported travel history within the assumed incubation period of 5 to 21 days. Of these, 20 cases travelled to Spain, where 16 attended an international pride event on Gran Canaria that took place from 5 to 15 May 2022.

Due to delay in diagnosis and reporting, further cases are to be expected in the last weeks depicted in the epidemiological curve.

From 23 May onwards, the outbreak started to gain momentum in Berlin and a shift to mainly autochthonous transmission occurred, with probable exposure to monkeypox virus (MPXV) in Germany (87%; 310/356) and particularly in Berlin (70%; 248/356) for the majority of cases. Overall, 66% of total cases (269/408) notified in Germany for whom PPOI was available reported Berlin as the PPOI. Most of them (93%; 249/269) also lived in Berlin.

## Epidemiological situation in Berlin

In the federal state of Berlin, the first case was reported to the LPHA on 20 May 2022. In response, the State Office for Health and Social Affairs in Berlin (SOHSA) together with LPHA immediately enhanced epidemiological surveillance by systematically interviewing cases using more specific questions to collect additional information, including whether cases identified themselves as men who have sex with men (MSM), possible exposures within Berlin (e.g. visit of clubs, bars) and stays outside of Berlin in the 21 days before symptom onset.

The following analysis is based on data reported and collected up to 21 June 2022. By this date, 353 MPX cases were reported in Berlin. Median time between disease onset and testing was 5 days (range: 0–20 days). Of 260 cases with available data, 259 identified themselves as being MSM.

**Figure 2:**
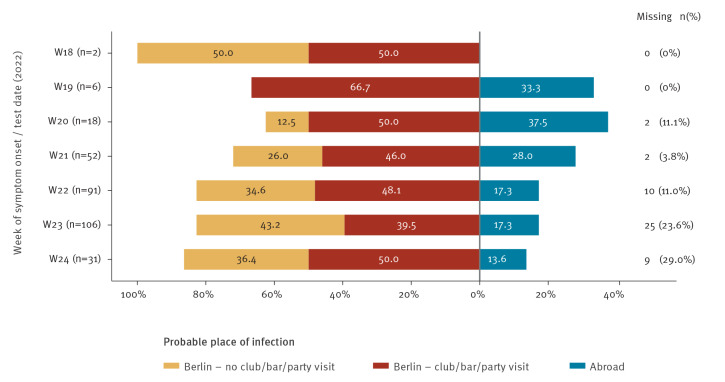
Probable place of infection of monkeypox cases by disease onset^a^ or test date^b^, Berlin, Germany, 20 May─21 June 2022 (n = 306)

PPOI: probable place of infection; w: week of reporting.

^a^ 295 cases with available information.

^b^ 11 cases with available information.

Information on the PPOI was available for 262 cases (74%). Of those, travel abroad within the incubation period was reported for 21% (n = 54). The most frequently mentioned travel destination was the international pride event on Gran Canaria (n = 15). Overall, 79% of cases (n = 208) reported no travel history and thus likely acquired MPX in Berlin and 58% of those cases (120/208) reported to have visited clubs, bars or private parties during their assumed infection period. Of note, in contrast to the cases notified by other German federal states, the majority of Berlin cases acquired their infection in Berlin in all weeks analysed. This includes the two cases in Berlin with the earliest date of symptom onset (2 May and 4 May 2022) in the outbreak. Over time, the proportion of cases with a PPOI in Berlin increased from 67% in week 19 to 86% in week 24, while conversely the proportion of cases reporting travel abroad as PPOI simultaneously decreased from 33% to 14% (Figure 2).

Cases with no information on PPOI (48/306) are denoted as missing on the right side of the figure. Cases with a PPOI in clubs and without travel history are denoted separately. Week 24 was incomplete at the time of writing.

## Discussion

The MPX outbreak in Germany so far has been centred in Berlin and mainly restricted to the MSM population. The outbreak in Germany is still ongoing and by 6 July 2022, a total of 1,304 cases have been notified to the RKI.

Early in the course of the outbreak, half of reported cases likely acquired their infection while being abroad; of these cases, many attended an international pride event on Gran Canaria. Notwithstanding, our data indicate that MPXV was concurrently circulating in Berlin before the outbreak was recognised, as two cases reported Berlin as their PPOI and symptom onset in the first days of May 2022, before the pride event took place. This is in line with the retrospective detection of cases in Portugal and the United Kingdom with disease onset as early as April 2022 [3-5]. From 23 May 2022 onwards, cases with autochthonous transmission were more frequent in Germany than travel-associated cases. Despite this shift, a notable proportion of cases in Berlin (> 10%) continued to acquire their infection abroad, indicating once again that the Berlin MSM community is intensely connected internationally and that controlling the outbreak requires a concerted effort of all affected countries [6].

Two thirds of cases in Germany reported Berlin as the likely place of infection. Berlin has one of the largest MSM populations in Germany and is a major international hotspot of the MSM community for visiting gay clubs, parties, and sex-on-premises locations [7]. Investigations of Berlin’s LPHAs indicate that a large proportion of autochthonous cases visited these locations during their assumed infection period, some more than once, suggesting the importance of these locations for disease transmission in Berlin.

The RKI and the SOHSA are cooperating with LPHA, sexual health clinics, MSM info points and community organisations to provide targeted information on MPX and to minimise further spread. In addition, RKI and the German Permanent Working Group of Competence and Treatment Centres for high consequence infectious diseases (STAKOB) provide information and training events for clinicians of interested Associations of the Scientific Medical Societies in Germany (AWMF) in order to provide them with updated epidemiological and medical knowledge on MPX on a regular basis. Information materials have been prepared in accordance with European Centre for Disease Prevention and Control (ECDC) guidance [8,9] and contain information on symptoms, transmission routes, and recommend those who develop symptoms to seek medical help and refrain from close contacts.

The Standing Committee on Vaccination (STIKO) in Germany currently recommends the use of the pox vaccine Imvanex (Bavarian Nordic, Hellerup, Denmark) for post-exposure prophylaxis after close-contact exposure to MPXV [10]. This includes close contact to non-intact skin or mucosa of MPX cases including sexual contact, close contact without suitable protective equipment in medical settings or contact to MPXV for laboratory personnel with unprotected handling of laboratory samples. Furthermore, vaccination is recommended as pre-exposure prophylaxis for persons with increased exposure and infection risk, including MSM ≥ 18 years of age with frequent change of sexual partners and, after individual risk assessment, personnel in specialised laboratories handling infectious samples. Imvanex is currently authorised in the European Union for the prevention of smallpox in adults and a review of data to extend the use for protection against MPX was started [11]. However, supplies are currently limited. Therefore, the European Medicines Agency recommends the import of the vaccine from the United States (US), where it is marketed under the brand name Jynneos (Bavarian Nordic, Morrisville, NC, US) and licensed also for the prevention of monkeypox.

## Conclusion

In order to effectively manage the MPX outbreak and reduce the risk of further spread, non-stigmatising, targeted information and recommendations for populations at risk, especially MSM [12], as well as the implementation of vaccination recommendations are necessary.
